# *NAMPT* haploinsufficiency is a therapeutic vulnerability to NAMPT inhibition in -7/-7q MDS

**DOI:** 10.1186/s40364-026-00956-6

**Published:** 2026-06-17

**Authors:** Nemo Ikonen, Tanja Ruokoranta, Salla Hyyppä, Ella Sinervuori, Juho J. Miettinen, Joseph Saad, Markus Vähä-Koskela, Caroline A. Heckman

**Affiliations:** 1 Institute for Molecular Medicine Finland - FIMM, Helsinki Institute of Life Science, University of Helsinki, Helsinki, Finland; 2 iCAN Digital Precision Cancer Medicine Flagship, University of Helsinki, Helsinki University Hospital, Helsinki, Finland; 3 Department of Hematology, Helsinki University Hospital Comprehensive Cancer Center, Helsinki, Finland; 4 Institute for Molecular Medicine Finland, University of Helsinki, Tukholmankatu 8, (P.O. Box 20), Helsinki, FI-00290 Finland

**Keywords:** MDS, NAMPT, Monosomy 7

## Abstract

Chromosome 7 abnormalities -7 and -7q define a high-risk subset of myelodysplastic syndromes (MDS) with poor prognosis. The *NAMPT* gene, located at 7q22.3, encodes a rate limiting enzyme (nicotinamide phosphoribosyl transferase) in the NAD+ salvage pathway. Several inhibitors of NAMPT have been developed, but their activity in MDS has not been previously described. In this study, we investigated if MDS myeloblasts are susceptible to NAMPT inhibition. We show that primary bone marrow cells from patients with -7/-7q MDS exhibit strong and select sensitivity. Bulk viability assays and single cell, multiparametric flow cytometry confirmed enhanced NAMPT inhibitor sensitivity across leukemic cell populations, especially CD34 + CD38+ blasts from -7/-7q MDS samples compared to non -7/-7q MDS and healthy donor bone marrow cells. The NAMPT inhibitor KPT-9274 combined with BCL2 inhibitor venetoclax was particularly effective at targeting MDS blasts compared to NAMPT inhibition alone. MDS samples with -7/-7q also showed significantly lower *NAMPT *expression compared to the non -7/-7q samples, indicative of haploinsufficient gene expression profile. In conclusion, these findings support *NAMPT* haploinsufficiency as a vulnerability and as biomarker for NAMPT inhibitor activity in -7/-7q MDS.

**Supplementary Information:**

The online version contains supplementary material available at 10.1186/s40364-026-00956-6.

**To the Editor,**

Monosomy 7 and deletion of the q arm of chromosome 7 (-7/-7q) are recurrent alterations in myelodysplastic syndromes (MDS) present in approximately 10% of patients, and are associated with poor prognosis, shorter survival, and increased risk of progression to acute myeloid leukemia (AML) [1, 2]. Nicotinamide phosphoribosyl transferase (NAMPT), encoded by the *NAMPT* gene at chromosome 7q22.3, is the rate limiting enzyme in the nicotinamide adenine dinucleotide (NAD+) salvage pathway [3]. NAMPT inhibitors have shown good efficacy in preclinical studies of hematological malignancies as they effectively target cancer cell metabolism by blocking the salvage pathway which leads to NAD+ depletion and cell death [4–7]. Previous studies have shown that AML blasts with -7/-7q are exceptionally sensitive to NAMPT inhibitors, with the sensitivity caused by *NAMPT* gene haploinsufficiency as a result of -7/-7q [8, 9]. The objective of this study was to investigate whether MDS blasts with -7/-7q are similarly susceptible to NAMPT inhibition, and if *NAMPT* haploinsufficiency could be a biomarker for NAMPT inhibitor activity in MDS.

The patient sample cohort is described in Supplemental Table 1, and the methods are described in full in the Supplemental Material and illustrated in Fig. 1A. We first assessed the bulk cell viability-based drug sensitivity screening results from bone marrow (BM) cell samples collected from patients with MDS and compared it to data from BM mononuclear cells (MNCs) from healthy donors that were published previously [8, 10]. MDS and healthy donor BM-MNCs were incubated with the NAMPT inhibitor daporinad for 72 h, and cell viability was measured using the CellTiter-Glo (CTG, -7/-7q *n* = 1, non -7/-7q *n* = 5, healthy *n* = 14) and CellTox Green (CTxG, -7/-7q *n* = 1, non -7/-7q *n* = 6, healthy *n* = 5) assays. Dose response curves (Fig. 1B–C) were summarized as drug sensitivity scores (DSS) (Fig. 1D–E, Supplemental Tables 2–3). Overall, MDS samples were significantly more sensitive to NAMPT inhibition compared to the healthy donor samples in the CTG assay (Supplemental Fig. 1A), indicative of an actionable therapeutic window and in line with previous studies in AML [4, 5, 8, 11]. The sample carrying -7/-7q was found to be the most responsive sample to NAMPT inhibition (Fig. 1D). A similar trend was observed in the CTxG assay, with the -7/-7q MDS sample being the most sensitive, but there was no significant difference between MDS and healthy donor samples (Fig. 1E, Supplemental Fig. 1B). To assess if the -7/-7q MDS samples express less *NAMPT* due to *NAMPT* gene haploinsufficiency, we analyzed the gene expression in CD34+CD38- cells which showed that samples with -7/-7q (*n* = 6) had significantly lower *NAMPT* expression compared to non -7/-7q samples (*n* = 24), indicating a haploinsufficient gene expression profile (Fig. 1F, Supplemental Table 4). Additionally, -7/-7q samples displayed significantly lower *NAMPT *gene expression compared to healthy samples (*n* = 6). The gating strategy for cell sorting is shown in Supplemental Fig. 2.

**Fig. 1:**
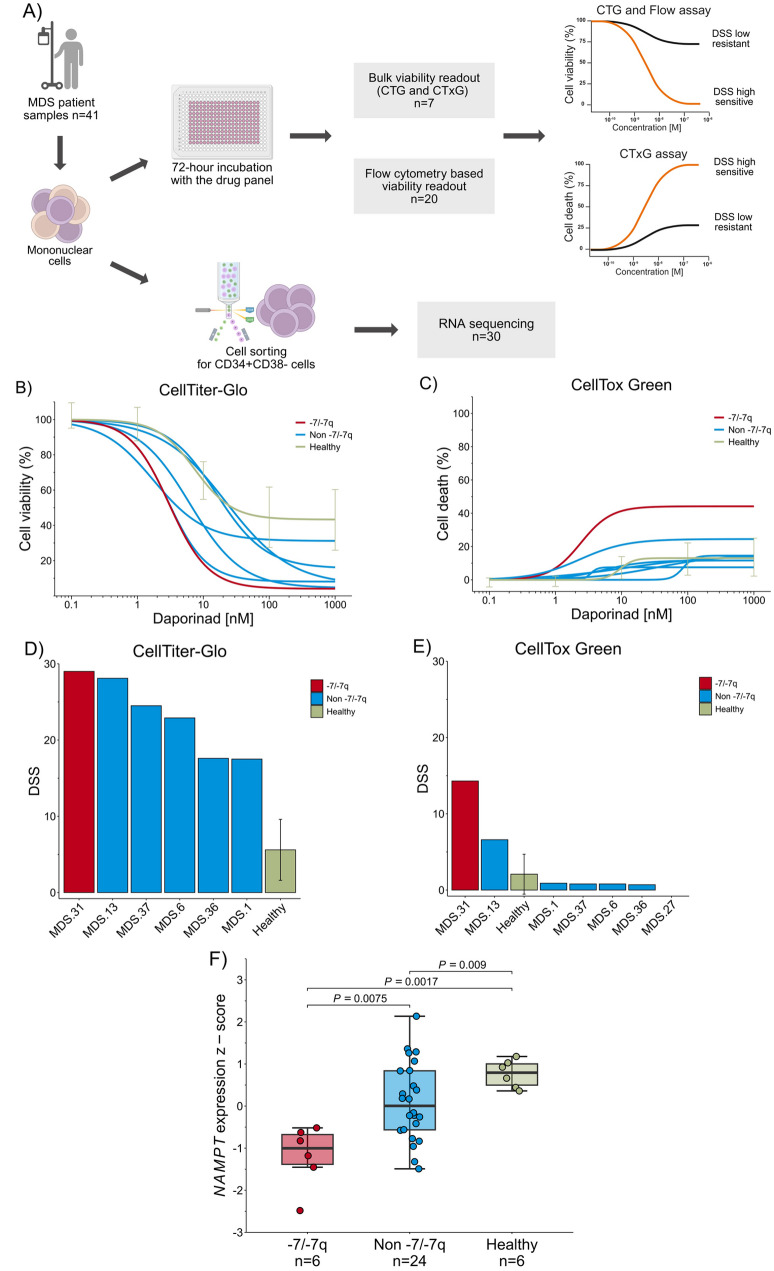
MDS samples are sensitive to NAMPT inhibition. (**A**) Illustration of the experimental workflow created with Biorender.com. (**B**) Dose response curves for CellTiter-Glo-based and (**C**) CellTox-Green-based bulk cell viability assays. Samples were treated with daporinad in 5 concentrations for 72 h. (**D**) Daporinad drug sensitivity score (DSS) values for MDS (*n* = 6) and healthy (*n* = 14) samples for CellTiter-Glo assay. Higher DSS indicates greater sensitivity. Data for healthy samples were summarized as one curve with standard deviations. (**E**) Daporinad DSS values for MDS (*n* = 7) and healthy (*n* = 5) samples for CellTox Green assay. Data for healthy samples (*n* = 5) were summarized as one score with standard deviation. (**F**) *NAMPT* gene expression z-scores were compared between -7/-7q (*n* = 6), non -7/-7q MDS samples (*n* = 24) and healthy samples (*n* = 6) using a two-sample t-test. *P* values were adjusted using the Benjamini-Hochberg method. Gene expression profiles were analyzed from RNA sequencing data of sorted CD34+CD38- cells

To investigate NAMPT inhibitor activity in different cell subpopulations, we performed flow cytometry-based drug sensitivity analysis. MDS BM-MNCs were incubated with the NAMPT inhibitors daporinad and KPT-9274 for 72 h (Supplemental Table 5) and subsequently stained with an antibody panel (Supplemental Table 6, Supplemental Fig. 3). We observed that NAMPT inhibitors effectively target the different myeloblast populations in MDS samples and that CD34+, CD117+, CD34+CD117+, CD34+CD38- and CD34+CD38+ blasts from -7/-7q MDS (*n* = 8) were significantly more sensitive to NAMPT inhibition by daporinad compared to non − 7/-7q MDS blasts (*n* = 12) (Fig. 2A, Supplemental Fig. 4, Supplemental Table 7). Similar trends were observed for KPT-9274, however, the results were not significant (Fig. 2B). Additionally, CD34+CD38+ blasts were more sensitive to daporinad and KPT-9274 than CD3+ T lymphocyte cells representing a healthy cell population control and CD14+ monocytic cell population in -7/-7q samples (Supplemental Fig. 5A-B). These results indicate that the cell populations with a leukemic phenotype, particularly CD34+CD38+, are sensitive to NAMPT inhibition.

**Fig. 2:**
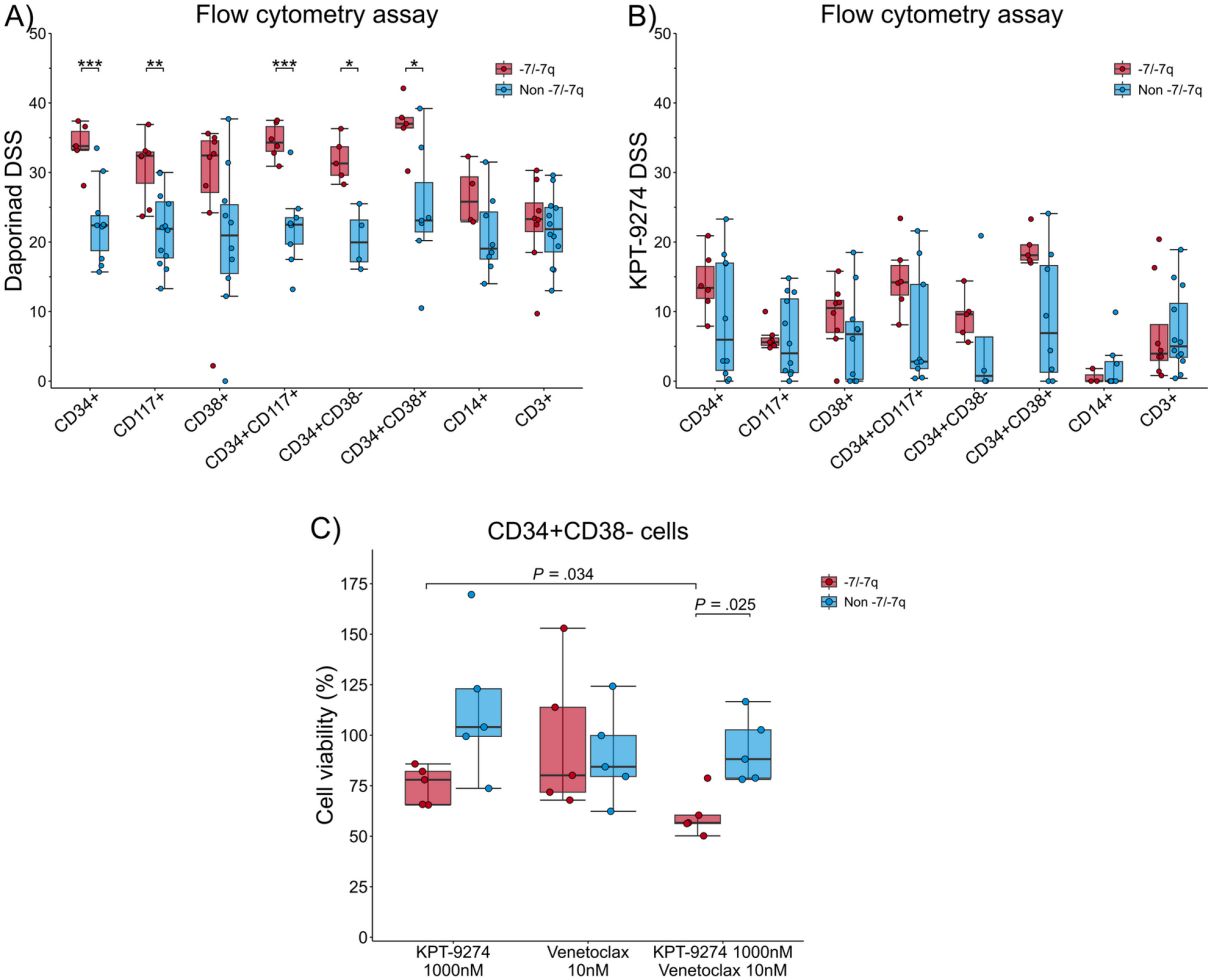
Flow cytometry-based drug sensitivity screening shows that MDS samples with -7/-7q are highly sensitive to NAMPT inhibition. Drug sensitivity scores for the different cell populations for (**A**) daporinad and (**B**) KPT-9274. MDS samples with (*n* = 8) and without (*n* = 12) -7/-7q were incubated with the inhibitors for 72 h and dose response curves were summarized as DSS values. Higher DSS indicates greater sensitivity. Samples with -7/-7q were compared to those without -7/-7q using a two-sample t-test. (**C**) Comparison of cell viability between single agents and the combination of 1000 nM of KPT-9274 and 10 nM of venetoclax in CD34+CD38- cells in -7/-7q (*n* = 5) and non -7/-7q (*n* = 5) MDS samples. Samples were treated with the inhibitors for 72 h, after which the cells were stained with detection antibodies to separate different cell types. Comparisons were done using a two-sample t-test and *P* values were adjusted using the Benjamini-Hochberg method

Next, we assessed KPT-9274 efficacy with three different clinically relevant drug combinations: venetoclax, azacitidine, and cytarabine. We observed that the combination of KPT-9274 and venetoclax was significantly more cytotoxic towards CD34+CD38- cells in samples with -7/-7q (*n* = 5) compared to KPT-9274 alone (Fig. 2C), and the combination was significantly more cytotoxic in -7/-7q compared to non -7/-7q samples (*n* = 5) (Fig. 2C). The addition of azacitidine or cytarabine did not significantly decrease cell viability (Supplemental Table 8, Supplemental Fig. 5C–D).

The data from this study demonstrate that NAMPT inhibitors are highly active in -7/-7q MDS ex vivo and establish *NAMPT* haploinsufficiency as a vulnerability and biomarker for NAMPT inhibitor activity. The findings suggest that patients with MDS, especially those with -7/-7q, could benefit from NAMPT inhibitors. As the number of samples included in our study was relatively small, the results should be interpreted with caution. Additionally, the lack of protein expression data does not allow us to validate haploinsufficiency at the protein level. However, these results support expanded investigations, including clinical evaluation of NAMPT inhibitors in high-risk MDS. 

## Supplementary Information

Below is the link to the electronic supplementary material.

Supplementary Material 1

Supplementary Material 2

## Data Availability

The data generated or analyzed during this study are included in this manuscript and its supplementary files. RNA counts data have been deposited to Zenodo:10.5281/zenodo.17091472. All other relevant data to the current study are available from the corresponding author (Caroline Heckman, caroline.heckman@helsinki.fi) on reasonable request.

## Abbreviations

MDS: Myelodysplastic syndromes
AML: Acute myeloid leukemia
-7/-7q: Monosomy 7 or deletion 7q
NAMPT: Nicotinamide phosphoribosyl transferase
NAD: Nicotine adenine dinucleotide
CTG: CellTiter-Glo
CTxG: CellTox Green
DSS: Drug sensitivity score
