# Supplementary material for: NAMPT haploinsufficiency is a therapeutic vulnerability to NAMPT inhibition in -7/-7q MDS
Source: Biomark Res. 2026 Jun 17;14:62. doi: 10.1186/s40364-026-00956-6 (PMC13274147; doi:10.1186/s40364-026-00956-6)

Supplementary Material

Supplementary methods

Patient samples

Bone marrow (BM) or peripheral blood (PB) samples were collected from patients with MDS after informed consent using protocols approved by the Institutional Review Board at the Helsinki University Hospital (permit numbers 239/13/03/00/2010 and 303/13/03/01/2011, Helsinki University Hospital Ethics Committee) in compliance with the Declaration of Helsinki. Mononuclear cells (MNCs) were isolated by Ficoll density gradient (Ficoll-Paque PREMIUM, GE Healthcare) and suspended in HS-5 derived conditioned medium (CM) (RPMI 1640, 12,5% HS-5 conditioned medium, 10% FBS, 2 mM-glutamine, 100 U/mL penicillin, and 100 µg/mL streptomycin). HS-5 derived conditioned medium production has been previously described[1]. Unless the sample was used immediately for drug sensitivity testing, the cells were viably frozen and stored in liquid nitrogen. Clinical and karyotype data was received from the Finnish Hematology Registry and Biobank to identify samples with monosomy 7 or deletion 7q. If karyotyping data was not available, whole exome sequencing data was used to determine the sample's chromosome 7 status.

Bulk *ex vivo* drug sensitivity testing

NAMPT inhibitor daporinad was dissolved in dimethyl sulfoxide (DMSO) and dispensed on 384-well plates (Corning) using Echo 550 Acoustic Dispenser (Beckman Coulter). Daporinad was plated in five different concentrations: 0.1, 1, 10, 100 and 1000 nM. Freshly isolated MNCs were plated on pre-drugged plates using 20 µL (10 000 cells) per well with the MultiDrop Combi (Thermo Scientific) peristaltic dispenser. Cells were incubated on the plates for 72 hours at 37°C in a humified incubator with 5% CO_2. After the incubation, cell viability or cytotoxicity was measured by adding CellTiter-Glo (CTG) or CellTox Green (CTxG) reagent (Promega), respectively, to the cells and the luminescence and fluorescence intensity was measured with the PheraStar FS plate reader (BMG Labtech). Viability measurements were normalized to negative (DMSO) and

positive (benzethonium chloride, BzCL) controls. Four-parameter dose response curves were fitted, and the drug sensitivity scores (DSS) were calculated as described previously[2].

Cell sorting

Frozen MNCs were thawed, suspended in CM and treated with Denarase (c-Lecta) for 30 minutes. The cells were then treated with ACK lysis buffer (150 mM NH_4Cl, 10 mM $KHCO_3$, 0.1 mM Na_2EDTA) for 5 minutes, spun down and resuspended in ice cold blocking buffer ((5 µL Human TruStain FcX plus (Biolegend), 5 µL True-Stain Monocyte Blocker (Biolegend), 90 µL FACS buffer (0.5% BSA in PBS)). Antibodies (**Supplemental Table 9**) were dispensed to 96-well conical bottom plates (Nunc, Thermo Fisher Scientific) using Echo 525 (Beckman Coulter). The cells were added to the plate and the plates were placed on a shaker for 20 minutes, after which the cells were washed and resuspended into FACS buffer. The cells were sorted for CD34+CD38- population using the BD Influx Cell sorter (BD Biosciences). The gating strategy is depicted in **Supplemental Figure 2**. Sorted cells were pelleted and resuspended into 700µL of Qiazol lysis reagent (Qiagen).

RNA sequencing and gene expression analysis

Total RNA was extracted from MNCs using the Qiagen miRNeasy kit or the Qiagen AllPrep kit. RNA quantification was done using the Qubit fluorometer (Thermo Fisher Scientific). Samples were sent for RNA sequencing in two batches. The first batch was sequenced by FIMM as follows: library preparation from 5 ng of total RNA was performed according to Takara SMARTseq v4 Ultra-low input RNA kit for Sequencing user manual (Takara Bio) followed by Illumina Nextera XT Library preparation according to Illumina Nextera XT Reference Guide (Illumina). Samples were sequenced on the Illumina NovaSeq6000 system using SP flow cell (Illumina) with read length for the single-end run was 75 bp. The second batch was sequenced by the Biomedicum Functional Genomics Unit as follows: library preparation from 2 ng of RNA was done with SMART-Seq® v4 Ultra® Low Input RNA Kit for Sequencing according to the user manual, followed by NEBNext® Ultra™ II FS DNA Library Prep Kit for Illumina. The samples were sequenced using NextSeq High Output with read length 75 bp. Reads were aligned to GRCh38.p13.

Raw counts were normalized using the Trimmed Means of M-values (TMM) method from the edgeR package and converted to log_2CPM[3]. Since the data was analyzed in two batches, the “removeBatchEffect” function from the limma package[4] was used to correct for technical batch effects. Corrected counts were converted to z-scores. Normalized and batch corrected values were used to compare the NAMPT gene expression between -7/-7q and non -7/-7q samples. The combined raw counts will be deposited in a public repository upon publishing.

Multiparametric flow cytometry-based *ex vivo* drug sensitivity testing

Daporinad, KPT-9274, azacitidine, cytarabine and venetoclax were dissolved in DMSO and pre-plated on 384-well conical bottom plates (Greiner) in up to 7 different concentrations (**Supplemental Table 5**) using Echo 550 (Beckan coulter) at High Throughput Biomedicine unit, FIMM. DMSO and BzCl were used as negative and positive controls. Frozen MNCs were thawed and resuspended in CM. Cells were first treated with Denarase to remove dead cell DNA and then left to recover from thawing for minimum 1 hour in CM. After recovery, the cells were treated with ACK lysis buffer for 5 minutes to remove red blood cells, then pelleted and resuspended in CM. The cells were plated on the pre-drugged 384-well plates at a volume of 20µl with either 10,000 or 30,000 cells per well, using the MultiFlo FX RAD dispenser (Agilent BioTek). The cells were incubated with the drugs for 72 hours at 37°C with 5% CO_2. Afterwards, the cells were stained with a detection antibody panel (**Supplemental Table 6**) using Echo 525. Cells were incubated with the antibodies for 30 minutes in the dark at room temperature. After incubation, the cells were analyzed on the iQue 3 or iQue Plus flow cytometers (Sartorius) using the Forecyt software (Sartorius). The gating strategy is shown in **Supplemental Figure 3**. Cell populations with <100 cells in DMSO negative control wells were removed from the analysis. Cell counts were normalized to the DMSO wells.

Statistical analysis

All statistical analyses were performed using R and R studio software[5,6]. Normality was assessed using the Shapiro-Wilk method. Comparison of two groups was done using either two-sample t-tests for normally distributed data or Mann Whitney U-tests for nonparametric distributions. Comparisons of multiple groups were done using Kruskal-

Wallis and Dunn Post-Hoc tests. The Benjamini-Hochberg method was used to adjust *P* values for multiple testing. The limit for statistical significance was set at *P* value or adjusted *P* value <0.05.

Citations for the supplementary methods

1. Karjalainen R, Pemovska T, Popa M, et al. JAK1/2 and BCL2 inhibitors synergize to counteract bone marrow stromal cell–induced protection of AML. *Blood*. 2017;130(6):789-802. doi:10.1182/blood-2016-02-699363

2. Yadav B, Pemovska T, Szwajda A, et al. Quantitative scoring of differential drug sensitivity for individually optimized anticancer therapies. *Sci Rep*. 2014;4:5193. doi:10.1038/srep05193

3. Robinson MD, McCarthy DJ, Smyth GK. edgeR: a Bioconductor package for differential expression analysis of digital gene expression data. *Bioinformatics*. 2010;26(1):139-140. doi:10.1093/bioinformatics/btp616

4. Ritchie ME, Phipson B, Wu D, et al. limma powers differential expression analyses for RNA-sequencing and microarray studies. *Nucleic Acids Res*. 2015;43(7):e47. doi:10.1093/nar/gkv007

5. R Core Team. R: A Language and Environment for Statistical Computing. Published online 2024. https://www.R-project.org/

6. Posit team. RStudio: Integrated Development Environment for R. Published online 2025. http://www.posit.co/

Supplementary Figure legends

Supplemental Figure 1. MDS samples are sensitive to NAMPT inhibition. A) Comparison of the Drug Sensitivity Score (DSS) values between MDS (n=6) and healthy samples (n=14) in the CTG -based cell viability assay. B) Comparison of the DSSs between MDS (n=6) and healthy (n=5) samples in the CTxG based cell viability

assay. Samples were treated with the NAMPT inhibitor daporinad in 5 different concentrations for 72 hours.

Supplemental Figure 2. Gating strategy for cell sorting to enrich CD34+CD38- cells from MDS patient samples. Viable single cells were gated using FCS, SSC and Helix Green viability marker. CD34+CD38- cells were gated from the CD45 dim cell population and sorted for RNA sequencing. Cells were sorted using the BD Influx Cell sorter.

Supplemental Figure 3. Gating strategy for flow-based drug sensitivity screening. Viable single cells were gated using FCS, SSC and the Annexin V and DRAQ7 markers. CD34+, CD117+, CD38+ and CD14+ cells were gated from the CD45+CD3- cell population. Cells were analyzed on the iQue Plus or iQue 3 flow cytometers and Forecyt software.

Supplemental Figure 4. Dose response curves for different cell populations from the flow cytometry-based drug sensitivity assay. Dose response curves for selected cell populations for NAMPT inhibitors A) daporinad and B) KPT-9274. Cells were incubated with the inhibitors in up to 7 increasing concentrations for 72 hours, after which the cells were stained with the antibody panel to distinguish the different cell populations. Viability markers Annexin V and DRAQ7 were used to separate dead and live cells.

Supplemental Figure 5. Comparison of the different cell population responses to NAMPT inhibition. Comparison of the cell population responses in -7/-7q samples to A) daporinad and B) KPT-9274. Cells were incubated with the inhibitors in up to 7 increasing concentrations for 72 hours, after which the cells were stained for flow cytometry assay with the antibody panel to separate the different cell populations.

Viability markers Annexin V and DRAQ7 were used to separate dead and live cells. Comparisons were done using Kruskal-Wallis and Dunn post-hoc tests. C) Comparison of cell viability between single agents and the combination of 1000 nM of KPT-9274 and 100 nM of cytarabine in CD34+CD38- cells in -7/-7q (n=5) and non -7/-7q (n=5) MDS samples. C) Comparison of cell viability between single agents and the combination of 1000 nM of KPT-9274 and 300 nM of azacitidine in CD34+CD38- cells in -7/-7q (n=5) and non -7/-7q (n=5) MDS samples. Samples were treated with the inhibitors for 72 hours, after which the cells were stained with antibodies to separate different cell types.

Supplemental Figure 1. MDS samples are sensitive to NAMPT inhibition due haploinsufficiency

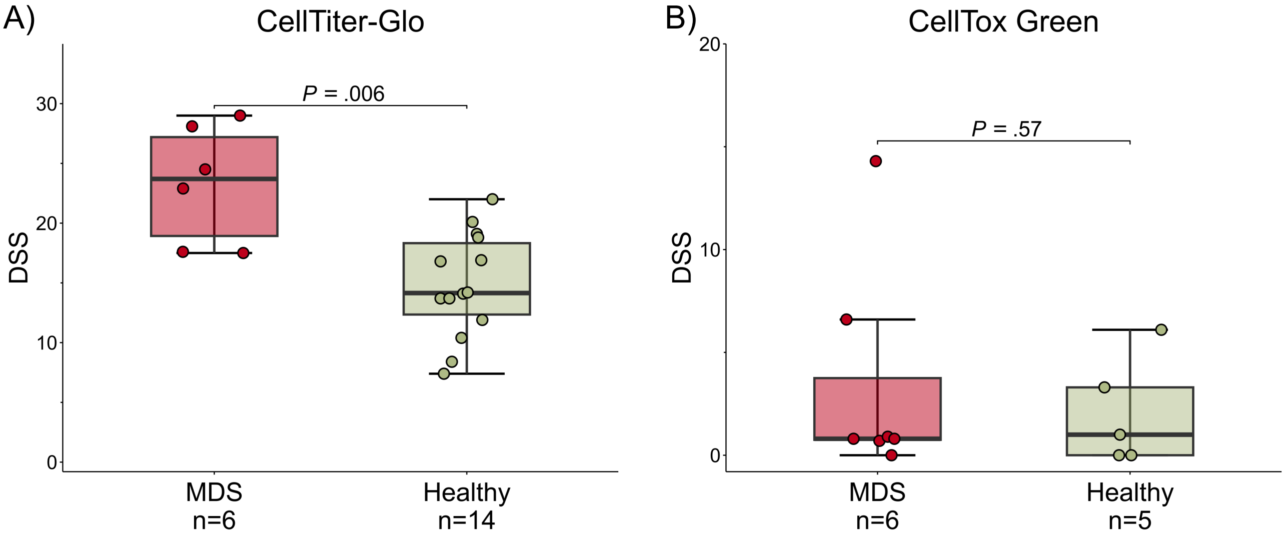

Supplemental Figure 2. Gating strategy for sorting CD34+CD38- cells

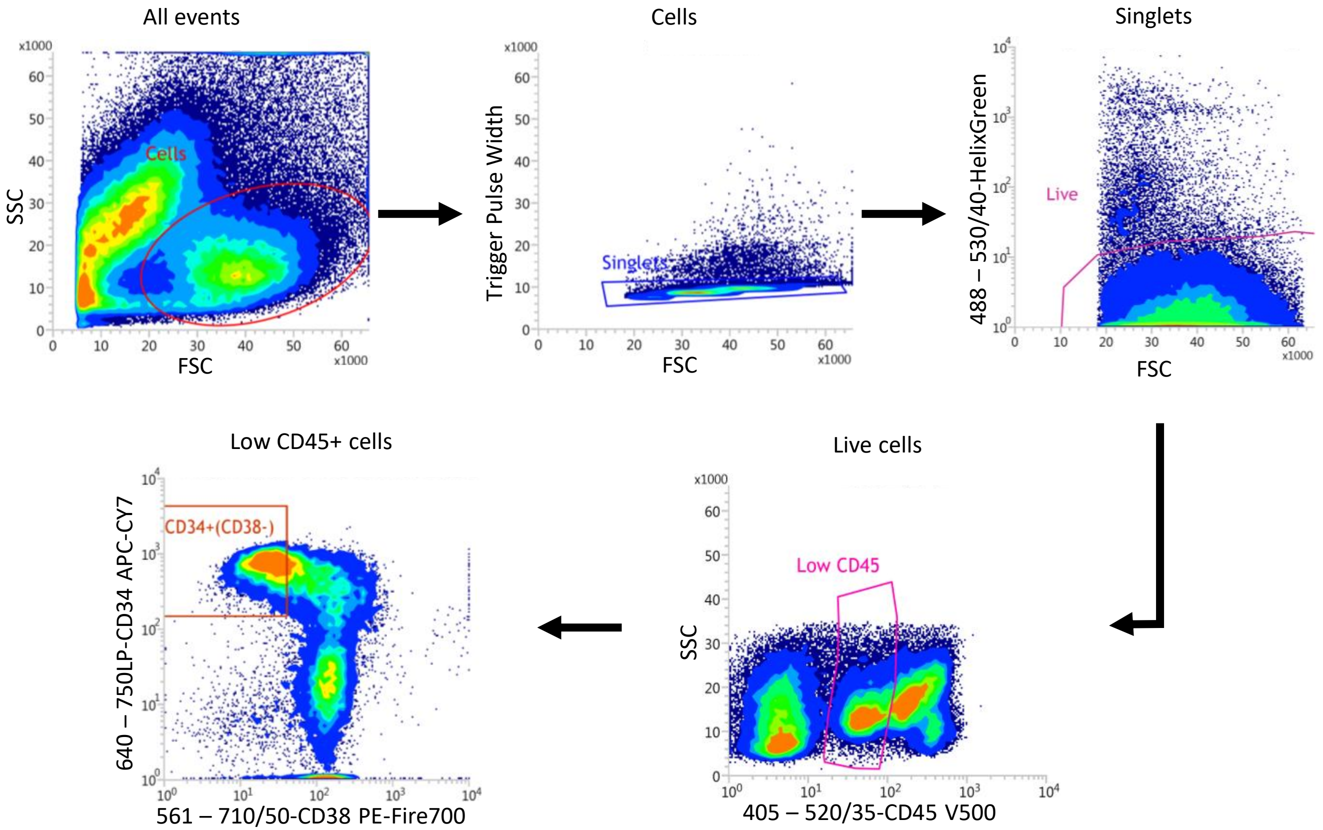

Supplemental Figure 3. Flow DSRT gating strategy

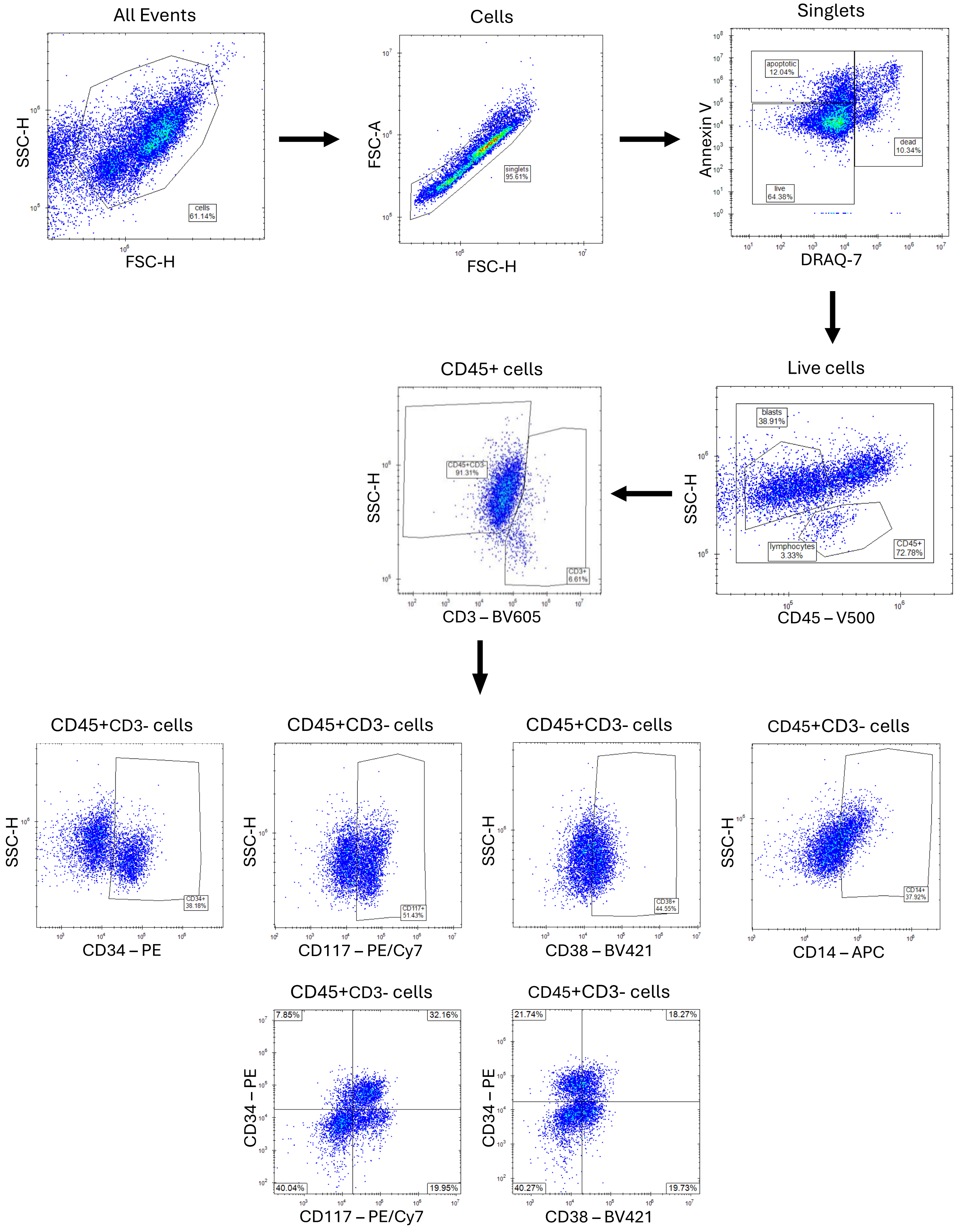

Supplemental Figure 4. Dose response curves for different cell populations from flow-based DSRT

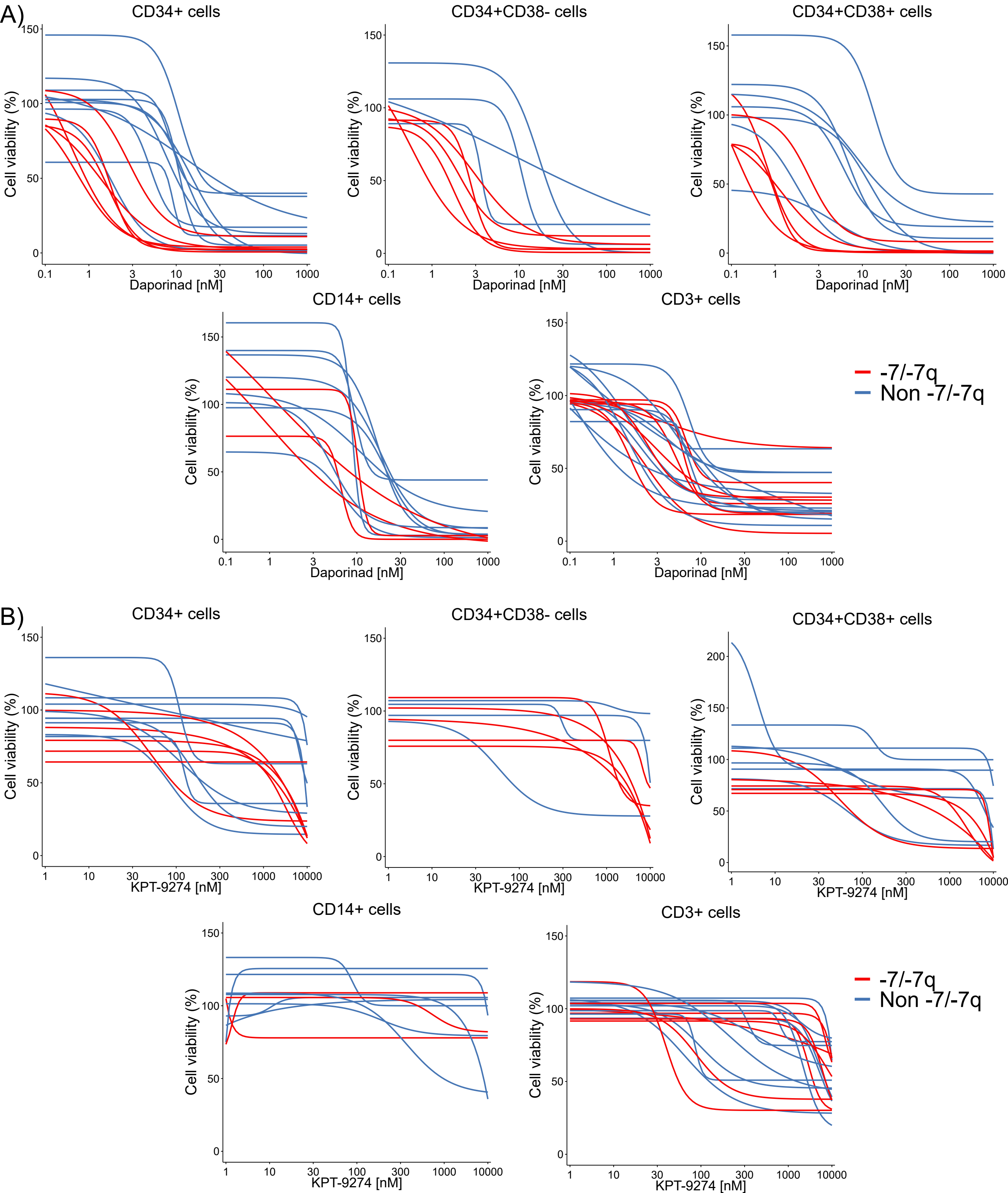

Supplemental Figure 5. Comparison of the different cell population responses to NAMPT inhibition

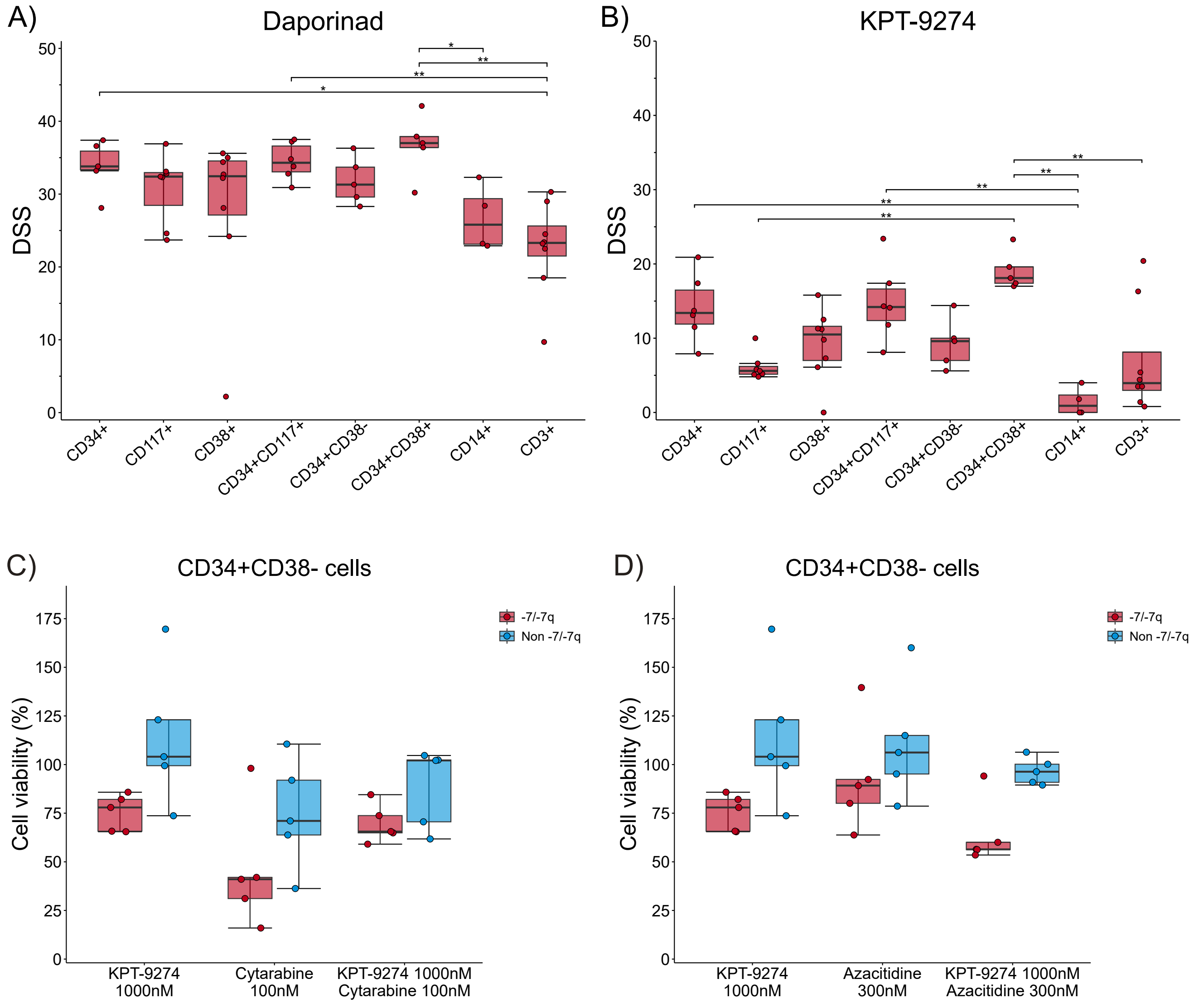

Supplement: Supplementary file 2 — Supplementary Material 2 [file 40364_2026_956_MOESM2_ESM.pdf]
